# Predicting the Risk of Intimate Partner Violence: The Chinese Risk Assessment Tool for Victims

**DOI:** 10.1007/s10896-012-9418-4

**Published:** 2012-03-10

**Authors:** Ko Ling Chan

**Affiliations:** Department of Social Work and Social Administration, The University of Hong Kong, Pokfulam, Hong Kong

**Keywords:** Validation, In-law conflict, Victimization, Receiver operating characteristic

## Abstract

The present study reports the development and validation of the Chinese Risk Assessment Tool for Victims (CRAT-V), an actuarial instrument for the prediction of intimate partner violence (IPV) victimization in a Chinese population. Data were collected from a representative sample of 2,708 Chinese women who were married or cohabiting in Hong Kong. All participants were interviewed with a questionnaire assessing their experience of IPV victimization and personal or family factors related to IPV. As measured by the Revised Conflict Tactics Scale (CTS 2), the base rates of preceding-year physical and sexual IPV victimization were 4.6 % and 3.6 %, respectively. Using a cross-validation procedure, the present study developed a 5-factor instrument with one half of the randomly split sample and validated the resulting tool with the other half. The CRAT-V had a sensitivity of 74.0 %, a specificity of 68.3 %, an overall accuracy of 68.7 %, and an area under the receiver operating characteristic (ROC) curve of 0.75 when administered on the second half of sample. Overall, the CRAT-V may serve as a straightforward, systematic, and easy-to-administer instrument tailor-made for Chinese populations for the assessment of risk of IPV victimization against women.

Intimate partner violence (IPV) has long been demonstrated to have serious harmful effects on its victims (Campbell and Soeken 1999; Leserman et al. 1998; Lindhorst and Oxford 2008; Yoshihama et al. 2009). Over the past few decades, there has been increasing attention given to various aspects of IPV worldwide (Garcia-Moreno et al. 2005; Krug et al. 2002). One aspect that has attracted growing interest is the assessment of the risk of IPV and its recidivism (Bennett Cattaneo 2007).

## Assessing IPV Risk

To facilitate the documentation and communication of IPV risk among professionals, different types of risk assessment tools or instruments have been developed. At present, one of the most commonly used risk assessments is clinical judgment, which usually relies on the exercise of professional discretion (Campbell et al. 2001; Dutton and Kropp 2000). Another commonly used approach is actuarial assessment, which predicts risk in both the absolute and relative sense by comparing the perpetrator or the victim to a norm-based reference group and providing an estimate of the probability of future violence (Kropp 2004). In general, assessment tools using this approach are usually completed by perpetrators, victims, and professionals lacking extensive training. All item ratings are weighted and summed into a total score, which serves as a basis for judging the level of violence risk. The revised Danger Assessment (DA; Campbell et al. 2009), the Ontario Domestic Assault Risk Assessment (ODARA; Hilton et al. 2004), and the Spousal Assault Risk Assessment Guide (SARA; Kropp et al. 1995; Kropp et al. 1999) are common examples of validated assessment tools utilizing the actuarial approach to help professionals assess IPV risk.

## Risk Factors for IPV

In order to facilitate the assessment and prediction of IPV, a better and clearer understanding of which factors affect IPV, and how these factors work, is essential. To date, numerous factors at the personal, family, and cultural level have been identified as associated with IPV risk. At the personal level, substance abuse (Caetano et al. 2005; Golinelli et al. 2009; Hien and Ruglass 2009; Logan et al. 2002), jealousy (Buss 2000; Wang et al. 2009), anger management skills (Barbour et al. 1998; Heru et al. 2007), violence approval (Hien and Ruglass 2009; Margolin et al. 1998; UNICEF 2009), dominance (Kim and Emery 2003; Straus 2006, 2008), stressful conditions (Cascardi and Vivian 1995; Neff et al. 1995), depressive symptoms (Danielson et al. 1998; Lehrer et al. 2006; Sugarman et al. 1996), sexual abuse history (Daigneault et al. 2009; Noll et al. 2003; Whitfield et al. 2003; Yoshihama et al. 2009), and experience of witnessing parental IPV during childhood (Cloitre 1998; Hien and Ruglass 2009; Yoshihama et al. 2009) were found to be significantly associated with the risk of IPV victimization. At the family level, relationship distress (Margolin et al. 1998; Stuart and Holtzworth-Munroe 2005; Vives-Cases et al. 2009), in-law conflict (Chan et al. 2009; Clark et al. 2010; Counts et al. 1999), and financial burden or indebtedness (Balmer et al. 2005; Chan et al. 2008; Kempson et al. 2004) are examples of factors related to IPV risk.

In addition, the concept of “face” may be one of the main cultural values that play a significant role in the risk of IPV in Chinese societies (Carr 1993; Hu 1944). According to Carr (1993), the complex Chinese term “face” can be translated as “prestige; dignity; honor; respect; and status” (p. 90) that can be gained or lost through interaction with others. Face has often acted as both a guideline for social behaviors and a product of social processes (Eberhard 1967; King and Myers 1977). Within a couple, face can be viewed as the reciprocated compliance, respect and/or defense that one expects from, and extends to, one’s partner. When face is “lost” (e.g., compliance or respect from the partner is reduced), anger and shame may be provoked. These feelings of anger and shame may then lead to the perpetration of IPV (Chan 2006).

Social desirability is a construct that is different from the concept of face. It may not be directly related to the risk of IPV, but rather to the reporting of IPV perpetration and victimization: the higher the level of one’s social desirability, the stronger the desire to be viewed positively and the lower the tendency for one to report experience of IPV (Arias and Beach 1987; Rosenbaum and Langhinrichsen-Rohling 2006). Researchers have suggested the inclusion of social desirability in IPV-related studies, so as to control for the accuracy of IPV reporting (Costa et al. 2007; Craig et al. 2006).

## The Present Study

Using a large and representative sample of Chinese households in Hong Kong, the present study aimed at developing and validating a brief and easy-to-administer IPV risk assessment tool for the Chinese population: The Chinese Risk Assessment Tool for Victims (CRAT-V). In order to design a tailor-made risk assessment tool specifically for Chinese female victims of IPV in Hong Kong, the present study incorporated several important characteristics of Chinese culture (e.g., “face” and the influence of in-law relationships) into the development of the CRAT-V. Despite the inclusion of culture-specific factors, the CRAT-V differs from other existing risk assessment tools by using risk factors to actually predict the likelihood of future IPV risk. At present, almost all IPV risk assessment tools evaluate one’s risk of future IPV recidivism by assessing the presence of IPV in the past (Campbell et al. 2009; Hilton et al. 2004; Kropp et al. 1995; Kropp et al. 1999). In contrast, the CRAT-V was designed to assess factors other than just one’s history of IPV experience. This makes the CRAT-V a more sensitive instrument that may be effective in avoiding underreporting caused by social desirability.

## Methods

### Sample

Data were obtained from a representative household population study conducted in 2004 in Hong Kong. A total of 4,347 eligible households were randomly sampled from the Register of Quarters maintained by the Census and Statistics Department of the Government of Hong Kong, which was the most up-to-date and complete sampling frame available. All family members of the selected households who met the inclusion criteria were invited to participate. The study criteria were: (a) 16 years of age or older, (b) Chinese ethnicity, (c) married or cohabitating, (d) ability to speak Cantonese, Mandarin or English, and (e) written informed consent. All eligible family members who had agreed to participate were interviewed face-to-face by trained interviewers. The study procedures were approved by the Ethics Committee of the University of Hong Kong. Of all eligible participants, a total of 5,049 Chinese adults were successfully interviewed, with a response rate of 70 %. The present study employed a sub-sample of data from the household population study. Only female respondents were included in the analysis procedures, giving a sample of 2,708 complete self-reporting records of married women.

### Measures

#### IPV Victimization

The validated Chinese version of the Revised Conflict Tactics Scale (CTS 2) was used to measure the experience of IPV victimization in the preceding year (Chan 2004). The CTS 2 covers five facets of spousal conflict: *negotiation*, *physical assault*, *psychological aggression*, *physical injury*, and *sexual violence*, and possesses satisfactory psychometric characteristics (Straus et al. 1996), high cross-cultural reliability (Straus et al. 1996), and satisfactory criterion validity (Coben et al. 1999). The internal consistencies of the CTS 2 subscales were good to excellent, with Cronbach’s alpha ranging from 0.79 to 0.95 in the initial study (Straus et al. 1996) and from 0.88 to 0.96 in the present study.

Only the subscales of physical assault and sexual violence were used in the present study. Respondents were asked if they were victims of these forms of IPV in the year preceding the interview. Any reported experience of IPV victimization was coded as “victimized by IPV.”

#### Personal and Relationship Profile (PRP)

The PRP is a self-report measure aimed at both clinical screening and research of domestic violence (Straus et al. 1999). The 21 subscales measure individual and relationship factors that are related to the etiology of IPV. The present study employed nine of those subscales, which are listed in Table 1 with brief descriptions and reliability coefficients. All items were rated on a 4-point Likert scale ranging from 1 (*strongly disagree*) to 4 (*strongly agree*) and were summed into a subscale score. In order to obtain a Chinese version of the PRP, a back-translation procedure was employed. The reliability coefficients of the Chinese PRP were moderate to high (Cronbach’s alpha = .60–.98).

**Table 1:** Brief descriptions and reliability coefficients of the selected subscales of the personal and relationship profile (PRP)

PRP subscale	No. of items	Alpha	Brief description
Anger Management	6	.60	Recognizing signs of anger, self-talk and behavioral self-soothing
Depressive Symptoms	8	.69	Disturbances in mood, dysphoric cognition and somatic disturbances
Domination	9	.95	Dominance describes relationships that are hierarchical and in which the person with greater advantage uses that advantage to gain status, privilege or control over his or her partner
Jealousy	8	.95	Extreme concern about the possible sexual and social exclusiveness of the current partner
Social Desirability	13	.60	The degree to which a respondent will tend to avoid admitting undesirable behavior, such as partner assault and other forms of crime
Stressful Conditions	8	.77	Stress or hassles experienced in daily living
Substance Abuse	7	.96	Excessive use of alcohol or other mind-altering drugs
Alcohol Abuse	3	.90	
Drug Abuse	4	.98	
Relationship Distress	8	.80	Areas of dissatisfaction with the relationship, characterized by high conflict and few positive interactions
Violence Approval	9	.82	The extent to which the use of physical force is acceptable in a variety of interpersonal situations

#### Face

The Acquisitive Face Orientation Scale (AFOS), a locally validated 10-item self-report scale, was employed to measure the concept of “face.” Respondents were asked to indicate whether a statement correctly described them on a 4-point scale ranging from 1 (*strongly disagree*) to 4 (*strongly agree*). The reliability coefficients of AFOS were 0.70 in a previous study and 0.93 in the present study, indicating a good internal consistency.

#### In-law conflict

Respondents were asked to report the frequency of conflict with their parents-in-law in the preceding year on an 8-point scale ranging from 0 (*never*) to 6 (*20 times or more*), with a code of 7 for “*none in the past 12 months, but it has happened before*.”

#### Childhood-Witnessed Parental Violence

Three subscales of the CTS 2 (physical assault, psychological aggression, and injury) were used to assess the experience of witnessing parental violence during childhood. The time frame of all items was modified and restricted to childhood. Any experience of witnessing IPV between parents reported by respondents was coded as having witnessed parental violence.

#### Demographic and Socioeconomic Characteristics

The Demographic Questionnaire was used to assess the demographic and socioeconomic characteristics of the respondents. Items covered age, education level, work status, income, indebtedness (i.e., whether they were in debt during the interview period), whether they had chronic illness, whether they had any disability, whether they were pregnant, whether they were new immigrants to Hong Kong, and whether they were receiving social security.

### Statistical Analyses

In order to develop a validated risk assessment tool, the split-half validation procedure was employed to cross-examine the accuracy of the newly developed instrument. The sample was split randomly in two: one for identification of significant predictors of IPV victimization and the other for cross-validation. With the first half of the sample, separate univariate logistic regression analyses were used to determine the odds ratios (*OR*s) for the association between the experience of IPV victimization and individual risk factors. All significant risk factors found were included in the subsequent multivariate stepwise logistic regression analysis, which gave the best set of predictors for IPV victimization. This set of factors was then validated with the second half of the sample, and the sensitivity, specificity, and overall accuracy was obtained for further comparison.

In addition, a Receiver Operating Characteristic (ROC) curve was compiled. The ROC curve is a graph plotting sensitivity against (1-specificity), and thus a graphical representation of the tradeoff between the positive and negative predictive values at every possible cutoff. The area under the curve (AUC), which is usually used to measure the accuracy of an assessment tool, was also examined. The AUC ranges from .50 to 1, and a higher value indicates a greater effectiveness of the assessment tool.

## Results

### Sample Characteristics and IPV Victimization

Table 2 shows a summary of the demographic characteristics and the rate of IPV victimization of the split samples. In the present sample of Chinese women, the preceding-year prevalence of physical and sexual IPV victimization was 4.3–4.6 % and 3.6–4.5 %, respectively. Results from the chi-square tests revealed no significant difference in the demographic profile or the IPV prevalence between the two randomly split samples.

**Table 2:** Demographic profile and prevalence of IPV victimization of the two randomly split samples

Characteristic	Percentage		
	1^st^ Half (*n* = 1,354)	2^nd^ Half (*n* = 1,354)	*χ* ^2^
Age group			1.84
18–25	1.8	1.3	
26–35	15.9	15.8	
36–45	31.7	30.8	
46–55	23.2	24.5	
56–65	12.8	12.8	
66 or above	14.6	14.9	
Chronic illness	20.3	21.5	0.57
Pregnancy/adoption/postnatal	2.1	2.9	1.84
Receiving social security	7.1	7.8	0.47
New immigrant of Hong Kong	8.0	6.9	1.05
Indebtedness	5.0	3.8	2.12
In-law conflict	4.8	4.6	0.07
Unemployed	3.5	3.5	0.01
Income group^a^			1.27
No income	54.3	52.3	
$4,999 or below	11.3	12.2	
$5,000 or above	34.4	35.5	
Disability	1.3	1.1	0.28
Substance abuse			
Alcohol abuse	5.0	4.9	0.03
Drug abuse	1.8	1.9	0.08
Preceding-year victimization			
Physical	4.6	4.3	0.16
Sexual	3.6	4.5	1.44

^a^In Hong Kong dollars (HKD). 1 HKD = 0.128 USD

*p < .05

### Selection of Factors for the CRAT-V

A series of univariate logistic regression analyses were performed, using one of the 15 potential risk factors as the predictor, and the experience of IPV victimization as the dependent variable (see Table 3). Of all of the factors, 13 had a significant *OR* (*p* < .05) and were thus included in the subsequent multivariate logistic regression analysis. Table 4 shows the set of factors in the final regression model, including jealousy, in-law conflict, sexual abuse history, stressful conditions, and relationship distress (all *p* < .01; Nagelkerke *R* ^2^ = .12). The five significant risk factors were grouped to form the CRAT-V.

**Table 3:** Odds Ratios (ORs) of the Risk Factors as Found with Univariate Logistic Regression Analyses (*n* = 1,354)

Risk Factor	Crude *OR*	(95 % CI)
Family factor		
Indebtedness	2.25*	(1.07, 4.71)
In-law conflict	3.74***	(1.95, 7.18)
Relationship Distress	3.43***	(2.00, 5.89)
Personal factors		
Substance abuse		
Alcohol abuse	1.66	(0.74, 3.76)
Drug abuse	2.60	(0.87, 7.78)
Domination	2.81**	(1.37, 5.77)
Jealousy	2.63***	(1.64 4.23)
Anger management	0.32***	(0.18, 0.58)
Violence approval	1.96*	(1.03, 3.72)
Depressive symptoms	3.79***	(1.95, 7.37)
Social desirability	0.14***	(0.06, 0.36)
Stressful conditions	2.92**	(1.52, 5.62)
Face	2.25**	(1.36, 3.72)
Sexual abuse history	7.75***	(3.33, 18.06)
Child witnessed parental violence	3.56***	(1.82, 6.98)

Dependent variable = Presence of preceding-year IPV (physical or sexual) as measure by CTS 2

*p < .05. **p < .01. ***p < .001

**Table 4:** The final multivariate stepwise logistic regression model (*n* = 1,354)

Risk Factors	B	S.E.	Wald χ^2^ (*df* = 1)	Odds Ratio (95 %CI)	Model LL	Change in -2LL
In-law conflict	1.12	0.36	9.79	3.06 (1.52, 6.17) **	−280.04	8.46
Relationship Distress	0.88	0.32	7.71	2.40 (1.29, 4.46) **	−279.56	7.50
Jealousy	0.79	0.26	8.99	2.20 (1.32, 3.70) **	−280.30	8.99
Stressful Conditions	0.89	0.40	5.02	2.44 (1.12, 5.33) *	−278.28	4.95
Sexual Abuse History	1.91	0.48	15.77	6.74 (2.63, 17.30) ***	−282.57	13.51
Constant	−8.44	1.18	51.03	–	–	–

Dependent variable = Presence of preceding-year IPV (physical or sexual) as measure by CTS 2

Nagelkerke *R* ^2^ of the final model = .12

*p < .05. **p < .01. ***p < .001

### Determination of the Optimal Cut-off Score

Table 5 shows the rates of hits, correct rejections, misses, and false alarms in both the first half and the second half of the split sample. The optimal cutoff probability, where the sensitivity and specificity values meet, was found to be 6.5 % in the present study. At this cutoff, sensitivity (the percentage of occurrences correctly predicted) was found to be 64 % (5 %/7.8 %), whereas specificity (the percentage non-occurrences correctly predicted) was 61.7 % (56.9 %/92.2 %). The positive predictive value (the percentage of correctly predicted occurrences) and the negative predictive value (the correct prediction of non-occurrence) were found to be 12.4 % (5 %/40.2 %) and 95.2 % (56.9 %/59.8 %), respectively. The overall accuracy for the correct prediction of both occurrence and non-occurrence was 61.9 % (56.9 % + 5 %).

**Table 5:** Rates of hits, correct rejections, misses and false alarms of the two split samples

Actual (%)	Predicted (%)				Total (%)	
	Did not Happen		Happened			
	1st Half	2nd Half	1st Half	2nd Half	1st Half	2nd Half
Did not Happen	56.9	63.0	35.3	29.3	92.2	92.3
	(Correct rejections)		(False alarms)			
Did Happen	2.9	1.9	5.0	5.7	7.8	7.7
	(Misses)		(Hits)			
Total	59.8	65.0	40.2	35.0	100.0	100.0

Based on a cut-off score of 6.5 %

Hits = Occurrence of incidents

Correct rejections = Non-occurrence of incidents

Misses = Unpredicted incidents (false negative)

False alarms = False prediction of incidents (false positive)

Sensitivity = Hits/Total happened = 5 %/7.8 % = 64 %

Specificity = Correct rejections/Total not-happened = 56.9 %/92.2 % = 61.7 %

Overall accuracy = Correct rejections + Hits = 56.9 % + 5 % = 61.9 %

To evaluate the trade-off between sensitivity and specificity over all of the possible cutoff probabilities of CRAT-V, a maximum likelihood estimate of the ROC using the present sample of female victims was obtained. The AUC with the present data was .70 (95 % CI = .64, .76), which was significantly greater than .50 under the 45° reference line (*p* < .001).

### Validation of the CRAT-V

The 5-factor CRAT-V was validated with the second half of the randomly split sample. The sensitivity and specificity of CRAT-V with the second half of the sample were 74.0 % and 68.3 %, respectively. In this case, CRAT-V had a positive predictive value of 16.3 %, a negative predictive value of 96.9 %, and an overall accuracy of 68.7 %.

A maximum likelihood estimate of the ROC using the second half of the randomly split sample of female victims was obtained. The AUC with the present data was .75 (95 % CI = .69, .81), which was significantly greater than 0.50 under the 45° reference line (*p* < .001).

## Discussion

Using a large and representative sample of the Chinese population, the present study undertook the development of the CRAT-V. The CRAT-V is a 5-factor, predominantly actuarial assessment tool for the evaluation of IPV risk in the Chinese population in Hong Kong. The CRAT-V performed well in distinguishing IPV victims from non-victims at a cutoff probability of 6.5 %, and achieved a fair AUC of 0.70 in the ROC analysis, providing supportive evidence for its validity.

The CRAT-V assessment tool possesses several advantages for the prediction of IPV in the Chinese population. First, the present study was conducted in China itself, while most research on IPV assessment tools has been performed exclusively in Western countries. The CRAT-V can thus be construed to be “tailor-made” and therefore of obvious practical value for the study of Chinese populations.

Second, the CRAT-V was developed and validated using a large and representative sample of the general Chinese population in Hong Kong. Although more supportive evidence is needed for its applicability to other Chinese populations worldwide (e.g., in China or the U.S.), this study provided preliminary evidence for its usefulness among a specific Chinese population (i.e., Hong Kong Chinese).

Third, the CRAT-V differs from other existing risk assessment tools by its use of factors other than past IPV experience. The less sensitive and crime-related items (e.g., jealousy and relationship distress) may reduce the impact of social desirability on reporting, and therefore increase the willingness of victims to disclose their IPV experience. Such a willingness on the part of the victims to report cannot be overemphasized as a critical feature of any assessment tool.

Finally, the predominantly actuarial CRAT-V was shown to be brief, straightforward, and simple to use. As a result, frontline service providers can utilize this new instrument without the need of any extensive knowledge of statistics or training for scoring and rating. In fact, the CRAT-V was designed in such a way that it allows self-reporting, so it is solely completed by the victims or suspected victims. In addition, the use of self-report may minimize the likelihood of any uninformed decisions made by professional judgment in the rating procedures (Dawes et al. 1989), and because of increased efficiency, the time and money costs incurred by a professional risk assessment may be reduced.

### Limitations and Suggestions for Future Studies

The present study took a retrospective approach and relied on the respondent’s memory. Any bias in a given victim’s recall and/or responses might be undetectable and yet might affect the results. A longitudinal prospective design might solve this problem; however, certain ethical issues must be addressed before one can launch a longitudinal study of the prediction of violence. One example is whether to provide for intervention in the predicted IPV cases. Future research must develop a better design by reducing the recall or response bias while at the same time minimizing any potential ethical problems.

Although the present study used a large, representative sample, all participants were residents in Hong Kong and this might limit the generalizability of the findings to other Chinese populations. Future research may validate the CRAT-V using Chinese populations in other cities or countries, such as cities in mainland China, Europe, and the United States. Also, as the concept of face may not be exclusive to Chinese populations (Goffman 1955), the application of the CRAT-V on populations other than Chinese may be feasible. Future research may also examine the applicability of the instrument in other populations than the ones just described.

The present study only used static linear predictive relationships and did not include any interaction effects in the prediction of IPV. There is evidence that risk factors of IPV, such as age and psychopathy (Harris et al. 1991), may interact. However, for simplicity and clarity, the present study did not include any interactive variable in the analyses. Future research should include interactive variables in the statistical analyses and evaluate whether the incorporation of such interaction effects would improve the predictive accuracy of the risk assessment tool.

The validity of the CRAT-V demonstrated in the present study may warrant the usefulness of cultural concepts or values in predicting the risk of violence. In addition to face, machismo may be one example of possible cultural-specific factors affecting IPV or its reporting. Machismo, most commonly shared by Latino populations, can be defined as values and behaviors associated with masculinity, invulnerability, and bravery (Whitaker and Reese 2007). In violence literature, it is also known as exaggerated hyper-masculinity expressed in terms of aggressiveness (Mosher 1991). Individuals with high level of machismo are supposed to be forceful, commanding, and decisive. Under the influence of machismo, IPV may not be perceived as a serious behavior that needs to be reported. Future research may incorporate machismo, or other cultural-specific concepts, in the development of IPV risk assessment tools to obtain more cultural-specific measures to predict violence risk.

## Conclusions

The present study developed and validated an actuarial risk assessment tool for in a Chinese population. This instrument adds a further piece to the growing body of evidence that supports the power of empirical methods in developing actuarial assessments for evaluating violence risk (Harris et al. 2002; Monahan 1996; Williams and Grant 2006). The CRAT-V is a straightforward, systematic, and easy-to-use instrument that has satisfactory predictive power for IPV in the Chinese population. Although it has been argued that actuarial assessments generally have the shortcoming of inflexibility for context-specific judgments (Kropp 2004, 2008), the present study provides evidence that the actuarial IPV risk assessment may be performed with a simple but reliable instrument for the prediction of IPV risk, without adding extra economic burden to public health providers.
